# Unraveling city-specific signature and identifying sample origin locations for the data from CAMDA MetaSUB challenge

**DOI:** 10.1186/s13062-020-00284-1

**Published:** 2021-01-04

**Authors:** Runzhi Zhang, Alejandro R. Walker, Susmita Datta

**Affiliations:** 1 Department of Biostatistics, University of Florida, 2004 Mowry Rd, Gainesville, FL 32610 USA; 2 Department of Oral Biology, University of Florida, 1395 Center Drive, Gainesville, FL 32610 USA

**Keywords:** Microbiome, OTU, WGS, Machine learning, Random Forest, Support vector machine, Linear discriminant analysis, PCoA, ANCOM

## Abstract

**Background:**

Composition of microbial communities can be location-specific, and the different abundance of taxon within location could help us to unravel city-specific signature and predict the sample origin locations accurately. In this study, the whole genome shotgun (WGS) metagenomics data from samples across 16 cities around the world and samples from another 8 cities were provided as the main and mystery datasets respectively as the part of the CAMDA 2019 MetaSUB “Forensic Challenge”. The feature selecting, normalization, three methods of machine learning, PCoA (Principal Coordinates Analysis) and ANCOM (Analysis of composition of microbiomes) were conducted for both the main and mystery datasets.

**Results:**

Features selecting, combined with the machines learning methods, revealed that the combination of the common features was effective for predicting the origin of the samples. The average error rates of 11.93 and 30.37% of three machine learning methods were obtained for main and mystery datasets respectively. Using the samples from main dataset to predict the labels of samples from mystery dataset, nearly 89.98% of the test samples could be correctly labeled as “mystery” samples. PCoA showed that nearly 60% of the total variability of the data could be explained by the first two PCoA axes. Although many cities overlapped, the separation of some cities was found in PCoA. The results of ANCOM, combined with importance score from the Random Forest, indicated that the common “family”, “order” of the main-dataset and the common “order” of the mystery dataset provided the most efficient information for prediction respectively.

**Conclusions:**

The results of the classification suggested that the composition of the microbiomes was distinctive across the cities, which could be used to identify the sample origins. This was also supported by the results from ANCOM and importance score from the RF. In addition, the accuracy of the prediction could be improved by more samples and better sequencing depth.

## Background

The advent of next generation sequencing (NGS) technologies for metagenomics has experienced a tremendous improvement, which allows the generation of large sequence datasets derived from diverse ecosystems, such as the human body, soil, and ocean water [1]. The use of whole genome sequencing (WGS) has been reported to have multiple advantages when compared with the 16S rRNA amplicon data [2]. As the composition of microbial communities can be location specific [3], studying the microbiome from different cities improves our understanding of city-specific microbes and their contributions to ecosystem composition and diversity.

This work could be regarded as a continuation of the work presented as part of the 2018 CAMDA MetaSUB challenge [4], we aimed to unravel city-specific signature and find the appropriate features for identifying and predicting the origin location of samples from different areas. In the 2018 CAMDA MetaSUB challenge, 12 cities were included with an unbalanced sample size design ranging from 5 to 60. By comparison, the current version data was much better with more cities included and more balanced sample size for each city. The main dataset covered 16 cities across the globe with sample sizes ranging from 10 to 26. Moreover, one dataset with 8 additional cities was provided as the mystery set. However, for the mystery dataset, the sample sizes for most of the cities were still limited with sample sizes of 6 out of 8 cities were below 10. The true city-information of the mystery data was provided much later in the process. All datasets in this work were provided by MetaSUB (http://camda2019.bioinf.jku.at/doku.php/contest_dataset), which aimed to build an international metagenomic map of urban spaces, based on extensive sampling of mass-transit system and other public areas around the world. They partnered with CAMDA for an early release of microbiome data obtained from global City Sampling Days, comprising the WGS metagenomics data. According to the open-reference picking, we included all OTUs with quality score greater than 0.5 (Please refer to Bioinformatics and data preparation section in the Methods section for more details). OTUs were aggregated as counts and selected taxonomic ranks, i.e. “order”, “family” and “species”, were used independently. Table 1 presented a tabulated insight of the data for all the cities.

**Table 1:** Number of samples included in the analyses and their corresponding city and country of provenance. Table also showed the number of “species”, “family”, and “order” existing in each city

City	Country	Number of samples	Number of samples after quality control	Species	Family	Order
**The main dataset**						
Auckland (AKL)	New Zealand	14	14	133	43	21
Berlin (BER)	Germany	21	21	235	101	51
Bogota (BOG)	Colombia	15	15	703	205	138
Hamilton (HAM)	New Zealand	16	16	141	39	22
Hong Kong (HGK)	China	18	17	289	112	60
Ilorin (ILR)	Nigeria	24	24	230	54	29
London (LON)	U.K.	24	22	48	29	15
Marseille (MAR)	France	10	10	212	85	44
New York (NYC)	U.S.A.	26	26	562	159	83
Offa (OFA)	Nigeria	20	20	349	85	42
Porto (PXO)	Portugal	20	20	286	122	66
Sacramento (SAC)	U.S.A.	18	18	554	208	126
Sao Paulo (SAO)	Brazil	24	24	227	99	54
Sofia (SOF)	Bulgaria	10	10	275	102	49
Stockholm (STO)	Sweden	20	20	186	76	42
Tokyo (TOK)	Japan	25	25	573	173	103
All cities	–	305	302	1047	276	180
**The mystery dataset**						
Brisbane (Bri)	Australia	7	6	74	53	32
Doha (Doh)	Qatar	3	3	59	38	24
Kiev (Kie)	Ukraine	8	7	144	80	45
Oslo (Osl)	Norway	12	12	505	148	83
Paris (Par)	France	8	6	136	73	41
Rio de Janeiro (Rio)	Brasil	12	12	343	109	53
Santiago (San)	Chile	6	6	309	113	58
Vienna (Vie)	Austria	5	5	167	72	35
All cities	–	61	57	700	188	109

## Results

### Selecting common features

As the composition of microbial communities can be location-specific, it would be helpful for us to unravel city-specific signature by investigating the composition of the common features. Based on the results from the open-reference OTU picking, 7 species, 9 families and 9 orders that existing across all the 16 cities were selected respectively. The number of features was limited and more information about the microbes was needed. To include more features, some additional rules were implemented: We ordered the “order”, “family” and “species” respectively based on their ubiquity across cities or samples and then selected the features with high ubiquity (Please refer to Selecting common features section in the Methods section for more details). By doing this way, more relative common “order”/“family”/“species” could be obtained respectively. The common features from different ranks were used for the analyses respectively. In addition, the combination of the common “species”, “family” and “order” were regarded as the combined features. By doing this, missing information of some non-common features was also included. For example, by using the features from the rank “family” only, the common “family” *Bacillaceae* was used for the analysis. However, by using combined features of ranks “order” and “family”, the corresponding “order” of *Bacillaceae*, i.e. *Bacillales*, would also be included. Therefore, more information about the non-common “family” belong to the order “*Bacillales*” would also be included even though with some information redundancy. Table 2 presented the details of the features selected based on additional rules. For simplicity, the mystery dataset was analyzed based on the common features and combined features. After common features were selected, the aggregated raw counts were normalized to log2-cpm for guaranteeing that counts were bounded away from zero to make the logarithm meaningful. Considering not only the structure of the data, but also the experimental design and number of samples, the different scale of the samples from different cities due to the technical variability could also be mitigated.

**Table 2:** The number of the features selected based on additional rules

Rules		Number of Features selected		
		Species	Family	Order
i) Features existing in at least N cities (Top features with the highest ubiquity across all the cities)	**The main dataset**			
	*N* = 15	13	23	17
	*N* = 14	26	31	19
	*N* = 13	52	43	23
	*N* = 12	75	54	29
	*N* = 11	110	64	33
	*N* = 10	150	73	36
	*N* = 9	188	86	43
	*N* = 8	234	97	48
ii) Top M features with the highest ubiquity across all the samples	M = 10	10	10	10
	M = 20	20	20	20
	M = 30	30	30	30
	M = 50	50	50	50
	M = 100	100	100	100
	M = 150	150	150	150
iii) Combination of the common features	“species”, “family” and “order”	25 (7 species, 9 families, 9 orders)		
	“species” and “family”	16 (7 species, 9 families)		
	“species” and “order”	16 (7 species, 9 orders)		
	“family” and “order”	18 (9 families, 9 orders)		
	**The mystery dataset**			
	“species”, “family” and “order”	41 (8 species, 18 families, 15 orders)		
	“species” and “family”	26 (8 species, 18 families)		
	“species” and “order”	23 (8 species, 15 orders)		
	“family” and “order”	33 (18 families, 15 orders)		

### Machine learning analysis

Different sets of features obtained were used for machine learning to find the set with the best performance on classification. For the main and mystery datasets respectively, three different classifications, i.e. Random forest (RF) [5], Support Vector Machine (SVM) [6] and Linear Discriminant Analysis (LDA) [7], with the leave-one-out cross-validation (CV) were implemented for all the selected feature sets. Each sample was selected in order to serve as the test sample with the remaining samples as the training dataset. The same training dataset was used for the three methods, and the same test sample was predicted by the three classifications. The results were recorded for each run. Table 3 presented the details of the classification error rate based on different rules using the main and mystery datasets respectively.

**Table 3:** The error rate with the leave-one-out cross-validation based on different rules. The number of features selected was retained in brackets

Methods	Random Forest			Support Vector Machine			Linear Discriminant Analysis		
Rules	Species	Family	Order	Species	Family	Order	Species	Family	Order
**The main dataset**									
**Common features**	0.588 (7)	0.306 (9)	0.253 (9)	0.571 (7)	0.296 (9)	0.270 (9)	0.615 (7)	0.323 (9)	0.340 (9)
**i) Features existing in at least N cities (Top features with the highest ubiquity across all the cities)**									
N = 15	0.459 (13)	0.365 (23)	0.375 (17)	0.463 (13)	0.365 (23)	0.355 (17)	0.512 (13)	0.372 (23)	0.379 (17)
N = 14	0.394 (26)	0.332 (31)	0.342 (19)	0.363 (26)	0.319 (31)	0.355 (19)	0.370 (26)	0.302 (31)	0.382 (19)
N = 13	0.359 (52)	0.292 (43)	0.295 (23)	0.356 (52)	0.302 (43)	0.295 (23)	0.353 (52)	0.286 (43)	0.294 (23)
N = 12	0.365 (75)	0.309 (54)	0.285 (29)	0.348 (75)	0.289 (54)	0.295 (29)	0.321 (75)	0.249 (54)	0.242 (29)
N = 11	0.360 (110)	0.296 (64)	0.295 (33)	0.333 (110)	0.282 (64)	0.291 (33)	0.323 (110)	0.256 (64)	0.219 (33)
N = 10	0.357 (150)	0.299 (73)	0.285 (36)	0.340 (150)	0.289 (73)	0.271 (36)	0.357 (150)	0.282 (73)	0.212 (36)
N = 9	0.317 (188)	0.292 (86)	0.311 (43)	0.317 (188)	0.302 (86)	0.281 (43)	0.393 (188)	0.262 (86)	0.199 (43)
N = 8	0.337 (234)	0.302 (97)	0.201 (48)	0.327 (234)	0.316 (97)	0.275 (48)	0.503 (234)	0.279 (97)	0.195 (48)
**ii) Top M features with the highest ubiquity across all the samples**									
M = 10	0.486 (10)	0.421 (10)	0.425 (10)	0.500 (10)	0.435 (10)	0.439 (10)	0.524 (10)	0.475 (10)	0.455 (10)
M = 20	0.385 (20)	0.328 (20)	0.341 (20)	0.381 (20)	0.351 (20)	0.338 (20)	0.388 (20)	0.318 (20)	0.321 (20)
M = 30	0.371 (30)	0.285 (30)	0.288 (30)	0.350 (30)	0.312 (30)	0.285 (30)	0.347 (30)	0.292 (30)	0.235 (30)
M = 50	0.291 (50)	0.309 (50)	0.271 (50)	0.288 (50)	0.286 (50)	0.265 (50)	0.271 (50)	0.256 (50)	0.195 (50)
M = 100	0.284 (100)	0.309 (100)	0.301 (100)	0.304 (100)	0.317 (100)	0.305 (100)	0.241 (100)	0.256 (100)	0.281 (100)
M = 150	0.283 (150)	0.312 (150)	0.308 (150)	0.297 (150)	0.336 (150)	0.348 (150)	0.303 (150)	0.292 (150)	0.411 (150)
**iii) Combination of the common features**									
7 species, 9 families, 9 orders	0.120 (25)			0.115 (25)			0.123 (25)		
7 species, 9 families	0.289 (16)			0.215 (16)			0.259 (16)		
7 species, 9 orders	0.210 (16)			0.189 (16)			0.237 (16)		
9 families, 9 orders	0.140 (18)			0.118 (18)			0.137 (18)		
**The mystery dataset**									
**Common features**	0.582 (8)	0.339 (18)	0.304 (15)	0.618 (8)	0.429 (18)	0.339 (15)	0.655 (8)	0.304 (18)	0.321 (15)
**iii) Combination of the common features**									
8 species, 18 families, 15 orders	0.268 (41)			0.339 (41)			0.446 (41)		
8 species, 18 families	0.375 (26)			0.464 (26)			0.411 (26)		
8 species, 15 orders	0.304 (23)			0.321 (23)			0.286 (23)		
18 families, 15 orders	0.250 (33)			0.339 (33)			0.339 (33)		

**Fig. 1:**
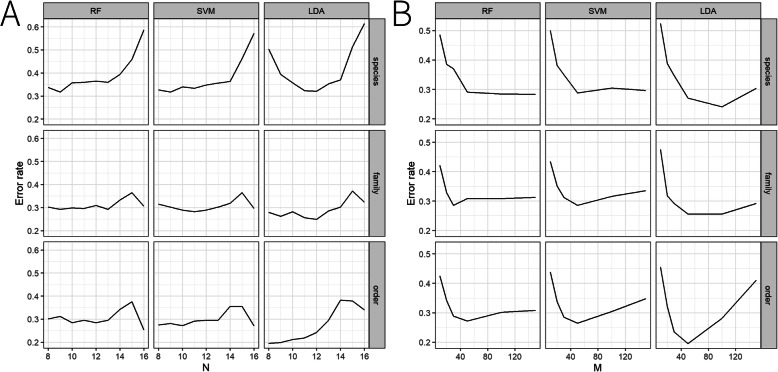
The changing trends of the error rate of different ranks and methods for the main dataset based on results of Table 2

Based on the results in Table 3, the changing trends of the error rate of different ranks and methods for the main dataset were presented in Fig. 1. As seen in Fig. 1a, when the features existing in at least N cities were used for the analysis, a decreased CV error rate was obtained for RF-species (i.e. qualitied “species” used for RF), SVM-species, LDA-species, LDA-family and LDA-order when decreasing the N (increasing the number of features). Furthermore, the lowest error rate was obtained by further decreasing the N. For RF-family and SVM-family, the error rate hasn’t changed considerably when we decreased the N at the “family” rank. Therefore, using the common “family” was better, as we obtained the low error rate without including too many features. In addition, for RF-order and SVM-order, the lowest error rate was obtained using the common “order”. Additionally, according to the Fig. 1b, when top M features with the highest ubiquity across all the samples were selected for analysis, error rates decreased with the increasing number of features used and then the lowest error rate was achieved, no matter which machine learning methods or which kinds of features we used for analysis. Moreover, for the combined features, the best performance was achieved using the combination of the common “species”, “family” and “order” (7 species, 9 families, 9 orders), the error rates obtained from RF, SVM and LDA were 12.0, 11.5 and 12.3% respectively, which were also the lowest among all the feature sets.

**Fig. 2:**
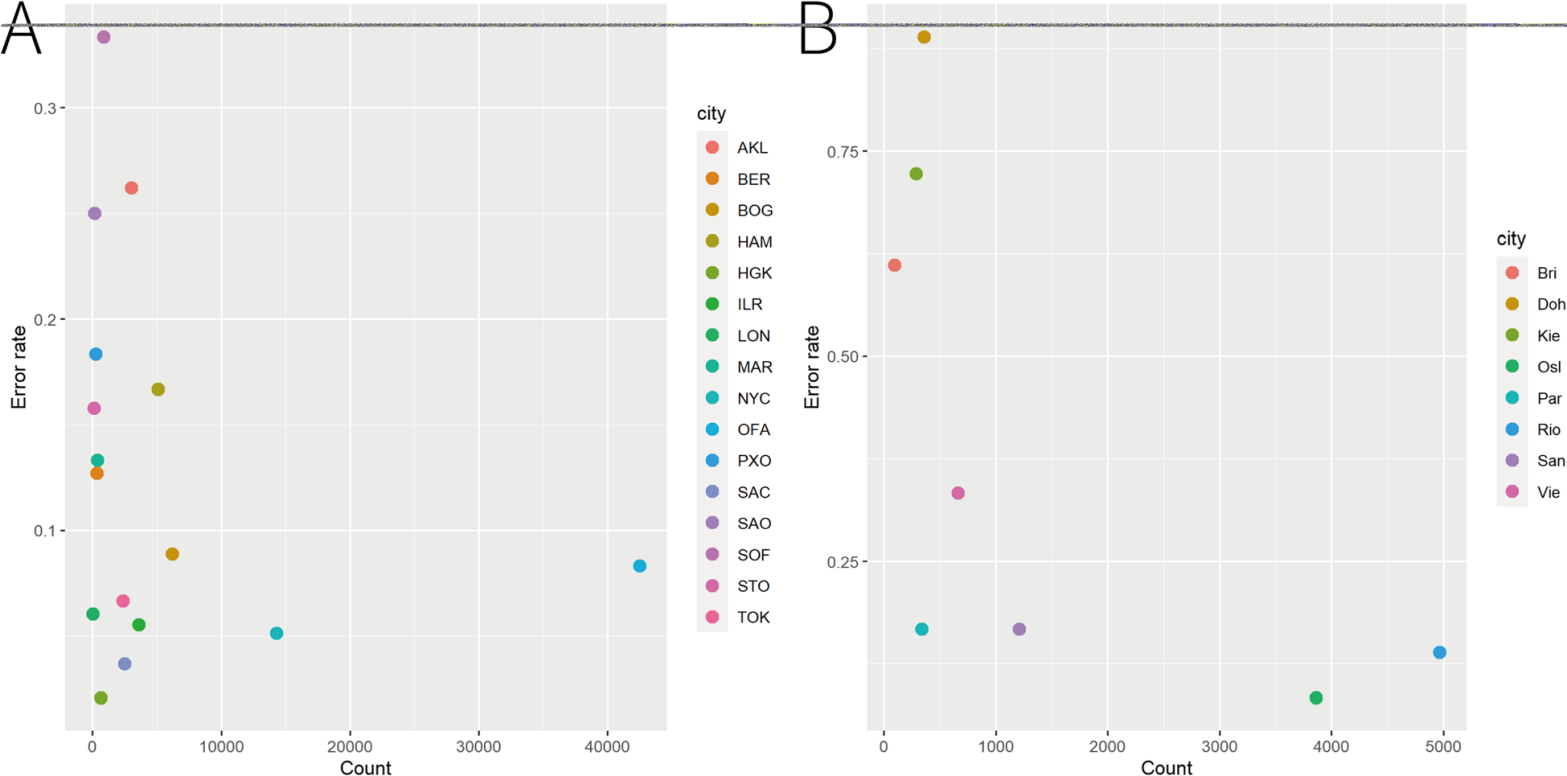
The association between the sequencing depth and the error rate, the x-axis is the sum of the count of the selected features, the y-axis is the error rate

The CV results using the feature set with the lowest error rate for the main dataset were presented in Table 4. It could be inferred from the Table 4 that for the cities with average error rate < 10%, most of them were with better sequencing depth such as Bogota, Ilorin, New York, Offa, Sacramento and Tokyo. The association between the sequencing depth and the error rate was visualized in Fig. 2. Regarding sequencing depth, we are referring specifically to the number of reads each sample has. Extremely low sequencing depth with only a few million reads could yield only a reduced number of OTUs. Correspondingly, having sequencing data with several hundreds of million reads is critical to obtain a decent OTU matrix. Therefore, we used the sum of the counts of the selected features based on the OTU matrix to represent sequencing depth for simplicity. From Fig. 2a, we could see that for the cities with high sequencing depth such as Offa and New York, consistent low error rates were obtained (8.33 and 5.13% respectively). For the other cities, the error rates varied considerably with the median error rate 14.56%. For example, Auckland and Hamilton, even though with good sequencing depth, their error rates were 26.19 and 16.67% respectively. By looking into the details of the results, we found that 3 samples in Auckland and 2 samples in Hamilton cannot be predicted correctly by any of the three methods. In other words, the microbial composition of these samples could be different from the other samples in the same city, making them difficult to be identified, which could be caused by the technical variability, as data were generated by many different people, different institutions. In addition, we found that London, the city with the poorest sequencing depth showed low error rate. Upon finding excessive zeros in samples from London, the samples of London could be easily identified from all the samples, which resulted in the low error rate of London. Therefore, the high discriminative power towards London samples could be stem from a technical artifact rather than a biological signal. Furthermore, according to the Table 4, some of the samples from other cities were predicted to London and another city with poor sequencing depth and high error rate, i.e. Sao Paulo, indicating that the sequencing depth of these samples could be as poor as samples from these cities, which resulted in misclassification for these samples.

**Table 4:** The cross-validation results using the feature set with the lowest error rate for the main dataset

True	AKL	BER	BOG	HAM	HGK	ILR	LON	MAR	NYC	OFA	PXO	SAC	SAO	SOF	STO	TOK
Predict																
**RF**																
AKL	11			1		1							1			
BER		19													1	
BOG			14								1					
HAM				14												
HGK					15											
ILR	1					23			1	1						
LON							18				1		2		2	
MAR								9								
NYC					1				25	1			1	1		1
OFA										18						
PXO							1				16		1			
SAC		1										18				
SAO		1	1	1			1				1		18	2		
SOF														7		
STO	1						1	1					1		16	1
TOK	1						1				1					23
Error rate (%)	21.43	9.52	6.67	12.50	6.25	4.17	18.18	10.00	3.85	10.00	20.00	0.00	25.00	30.00	15.79	8.00
**SVM**																
AKL	11			1		1				1			1			
BER		17						1			2	1			1	
BOG			14													
HAM	1			13												
HGK					16											
ILR		1				23			1							
LON							22				1		2		2	
MAR								8								
NYC				1					25				1			1
OFA										19				1		
PXO			1					1			16		1			
SAC												17				
SAO		1		1							1		19	4		
SOF		1												5		
STO	1							1					1		16	
TOK	1	1														24
Error rate (%)	21.43	19.05	6.67	18.75	0.00	4.17	0.00	20.00	3.85	5.00	20.00	5.56	20.83	50.00	15.79	4.00
**LDA**																
AKL	9			1									1			
BER		19		1							1		1		1	
BOG			13													
HAM				13												
HGK			1		16					1						
ILR						22			1					1		
LON	1						22				2		4		2	
MAR								9	1							
NYC									24							1
OFA						2				18						
PXO	1			1				1			17					
SAC	1											17				
SAO	1		1									1	17	1		
SOF										1			1	8		
STO															16	1
TOK	1	2														23
Error rate (%)	35.71	9.52	13.33	18.75	0.00	8.33	0.00	10.00	7.69	10.00	15.00	5.56	29.17	20.00	15.79	8.00
**Three methods**																
City	AKL	BER	BOG	HAM	HGK	ILR	LON	MAR	NYC	OFA	PXO	SAC	SAO	SOF	STO	TOK
Average error rate (%)	26.19	12.69	8.89	16.67	2.08	5.56	6.06	13.33	5.13	8.33	18.33	3.70	25.00	33.33	15.79	6.67

The same procedures were implemented for the mystery dataset, the lowest average error rate was obtained when we used the combined features with 8 species and 15 orders. The error rates were 30.4, 32.1 and 28.6% for RF, SVM and LDA respectively. Compared to the results of the main dataset, the higher error rate was obtained using the mystery dataset, even though with less cities included. The possible reasons could be the limited sample size and the poorer sequencing depth for the whole mystery dataset. As seen in Fig. 2b, the cities with a high sequencing depth including Rio de Janeiro and Oslo showed the lower error rates (13.89 and 8.33% respectively) among all the cities. For the other cities, the high variability of error rates was also observed with median error rate 47.22%. The evidence from Fig. 2 that cities with high sequencing depth had consistent lower error rates indicated the necessary of deep sequencing for successfully predicting the provenance of samples.

Additionally, to test the consistency of the performance, 20% of the samples from each city in the main dataset were randomly selected as the test set, the remaining samples were used to do the leave-one-out CV and predict the labels of the test samples using the feature set with the lowest error rate. The CV error rates of leave-one-out for the three methods were 13.33, 13.75 and 13.75% respectively, and the test error rates were 11.67, 10 and 15%, which were close to our previous CV results.

In addition to the analyses based on the main and mystery datasets respectively, we have used the models built based on the main dataset and part of the mystery dataset to predict the remaining samples from the mystery dataset. Knowing that the main dataset and the mystery dataset had no cities in common, using the main dataset only to correctly predict the samples from the mystery dataset was impossible due to a lack of information about the mystery dataset. Therefore, 50% of the samples of each city from the mystery dataset were randomly sampled and added to the main dataset to serve as part of the training dataset, the remaining mystery samples were used as the test samples. In the training dataset, the samples from the mystery dataset were given a new label “mystery”. The feature set used for the classifications were the common “family” (5 families) and “order” (6 orders), as there were no common “species” between the main and mystery dataset. Random samplings along with the three different methods were conducted for 1000 times independently. The test samples were correctly predicted as the “mystery” with the average error rates of 10.48%, 9,21 and 10.36% for RF, SVM and LDA respectively, indicating that the mystery samples could be effectively identified from the samples of main dataset. In other words, although with the limited information about the mystery samples, we could still make the inference if the mystery samples belong to the cities we have in our training model.

The following analyses were based on the feature set with the lowest error rate.

### Principal coordinates analysis

**Fig. 3:**
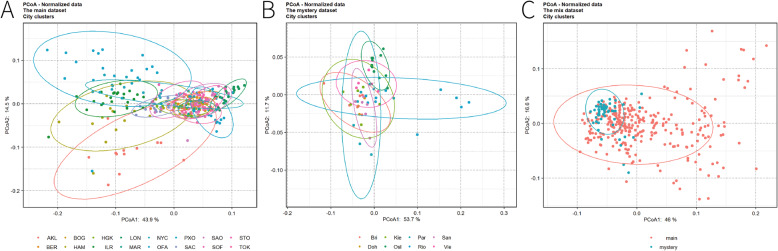
The PCoA plot with the first and the second axes: main dataset in **a** and mystery dataset in **b**

The results of PCoA [8] were presented in Fig. 3. Figure 3a illustrated the main dataset with 58.4% of total variability of the data explained by the first two PCoA axes. A separation of the cities could be referred from the plot. Specifically, London was separated from most cities and on the rightmost site, which was corresponding to the results from machine learning methods, as the poor qualify of samples from London made them different from most of the samples. In addition, the samples from Ilorin and Offa (both the cities of Nigeria) were away from the most samples and showed a massive overlap in the upper left corner, which was corresponding to the results of Table 4 that some of the samples from these two cities were predicted to each other. Furthermore, more overlaps were observed among the other cities, making them more difficult to be identified. The result of the mystery dataset was given in Fig. 3b. The first two PCoA axes explained 65.4% of the total variability of the data, which was comparable with the percentage explained in the main dataset. Although many cities overlapped, samples of Oslo were clustered together and distributed at the top of the plot, separating from the most samples. This was also corresponding to the low error rate from the previous analysis. Moreover, the Fig. 3c presented the PCoA based on the mix of the main and mystery datasets, we could see that most of the samples from the mystery dataset located at the left side of the plot, however, the separation between the two datasets was not obvious.

### Analysis of composition of microbiomes

**Fig. 4:**
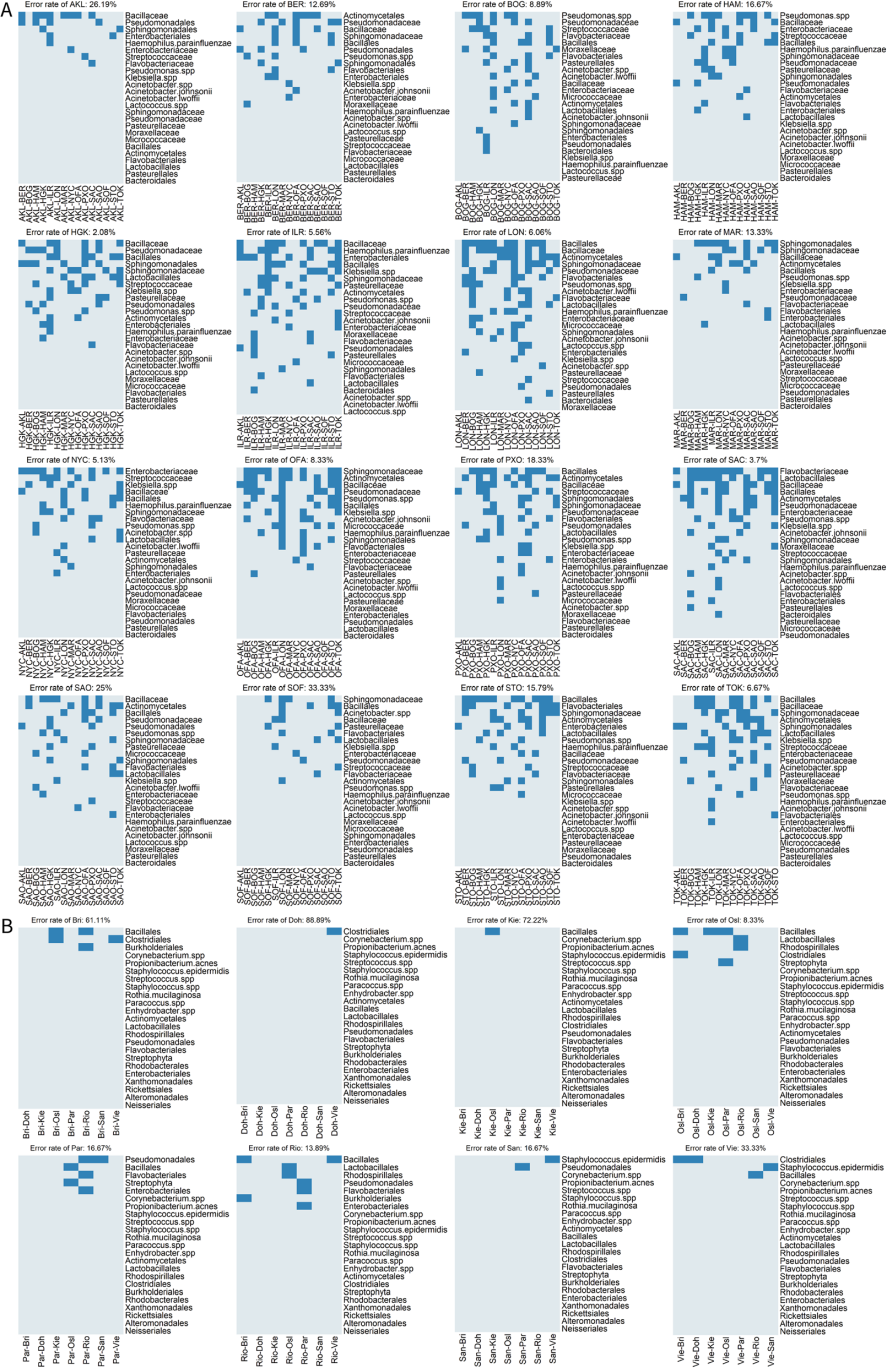
The analysis of composition of microbiomes across all pair-wise comparisons of cities: main dataset in **a** and mystery dataset in **b**. The significant features are denoted by deep blue, the features that are not significantly different in two cities are denoted by light blue

**Fig. 5:**
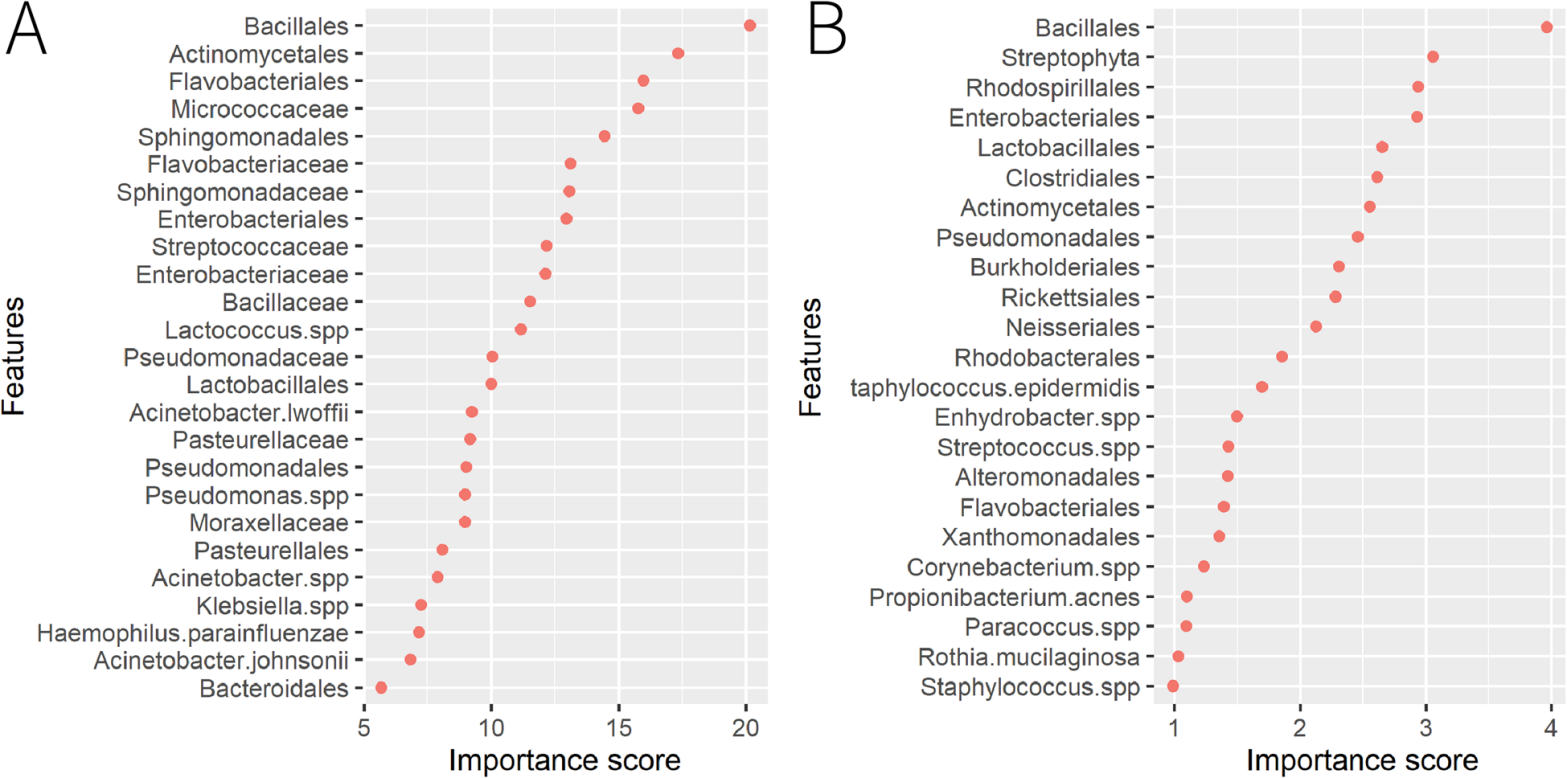
The importance of features obtained from the RF: main dataset in **a** and mystery dataset in **b**. The features are ordered by the importance

The results from the analysis of composition of microbiomes (ANCOM) [9] were presented in Fig. 4. The relative abundances of the features were used to conduct the pair-wise comparisons among all the cities. Upon the significance of the features, the differentially abundant features were found. The features on the right were ordered by the number of times the relative abundance was significantly different in the pair-wise comparisons. As presented in Fig. 4a, *Bacillaceae*, *Bacillales*, *Actinomycetales*, *Sphingomonadaceae*, *Pseudomonadaceae*, *Pseudomonas.spp*, *Sphingomonadales*, *Lactobacillales*, *streptococcaceae* and *Enterobacteriaceae* were the top 10 features with the highest counts among all the comparisons for the main dataset. Interestingly, the top 1 significant feature for the Sacramento, i.e. *Flavobacteriaceae*, which was found to be significantly different in 12 out of 15 comparisons, was not in the top 10 features, indicating the uniqueness of *Flavobacteriaceae* for Sacramento as it could be the city-specific signature, helping identify the Sacramento samples from other samples. In Fig. 4b, the top 10 features were *Bacillales*, *Clostridiales*, *Pseudomonadales*, *Staphylococcus.epidermidis*, *Lactobacillales*, *Rhodospirillales*, *Flavobacteriales*, *Streptophyta*, *Burkholderiales* and *Enterobacteriales* for the mystery dataset. Little change was seen from the importance score order (Fig. 5) derived from the Random Forest. It could be inferred from Fig. 5a that *Bacillales, Actinomycetales*, *Sphingomonadales*, *Sphingomonadaceae*, *Enterobacteriaceae* and *streptococcaceae* were also in the top 10 features. Similarly, for the mystery dataset, *Bacillales*, *Streptophyta, Rhodospirillales*, *Enterobacteriales*, *Lactobacillales*, *Clostridiales* and *Pseudomonadales* were in the top 10 of both lists. In summary, the common “family” and “order” of the OTU provided the most informative data for predicting the origins of the samples from the main dataset, which was also corresponding to the results of machine learning that the error rate of using common “family” or “order” only was much lower than the error rate of using common “species”. For the mystery dataset, the common “order” dominated the prediction.

## Discussion and conclusions

For the CAMDA challenge MetaSUB data of this year, 16 cities were included and 10 or more samples were collected for each city in the main dataset. Selecting common features, normalization, three methods of machine learning algorithms, PCoA and ANCOM were conducted for both the main and mystery datasets.

Common features selecting along with the machine learning methods showed that the combination of the common features could be the effective microbial fingerprint for unraveling city-specific signature and identifying sample origin locations. Therefore, more taxonomic ranks of microbiomes such as “genus” could be added to the combination to investigate the performance of the prediction in the future works. Using the common features may help us to unravel the composition of microbial community of each city. In addition, by using the common features, the low error rates were obtained without including too many features for both datasets. However, some city-specific features might be ignored by doing this way as these features existed in a specific city and will be removed by the procedure. In our previous CAMDA experience, we have tried to select sets of data focusing on the city-specific features that were differentially present across the cities, the resulting data was loaded with lots of zeros that some analyses such as PCA and variance estimations were not at all behaving in a good manner. It’s worth investigating to see whether combining the common features with city-specific features will provide a more powerful prediction in our future work, as including the city-specific features provides us more information about the specific city.

Additionally, according to the results from machine learning, the cities with high sequencing depth generally seem to have lower error rate, indicating the necessity of the decent sequencing depth for the prediction. However, for the cities with relatively poor sequencing depth, the error rates varied considerably. For example, on one hand, cities with poor sequencing depth such as Sofia and Sao Paulo in the main dataset and Doha, Kiev and Brisbane in the mystery dataset were showed to have high error rates. On the other hand, for cities such as Hong Kong in the main dataset and Paris in the mystery dataset, low error rates were obtained even though with poor sequencing for these cities. The high variability in error could be caused by other sources rather than the low coverage such as technical variability, as the data was generated by different institutions from different countries. Therefore, in addition to improving the sequencing depth, the prediction could be further improved by reducing the possible technical variability to make the data generation unified. Totally eliminating the technical variability could be a hard task, however, by doing the normalization, technical variability between different cities such as different scales could be mitigated to some extent. Additionally, the difference of the microbial composition of samples within the same city, such as samples from Auckland and Hamilton, could also be caused by the technical variability, which hinders the ability of classifier for predicting.

Moreover, we have used the main dataset and half of mystery samples as our training dataset to predict the labels of another half of mystery samples. The error rates were 10.48, 9.21 and 10.36% for RF, SVM and LDA respectively, most of the samples from mystery dataset could be identified correctly by the classification.

PCoA analysis for both datasets showed that nearly 60% of the total variability of the data could be explained by the first two PCoA axes, most cities overlapped with each other. However, some cities such as Offa, Ilorin and Oslo were separated from most cities, indicating the unique composition of microbiomes in these cities, which was corresponding to the low error rates in machine learning analysis.

The heatmaps of ANCOM revealed that some of the features, such as the common “family” and “order” in the main dataset and the common “order” in mystery dataset, were significantly different in pair-wise comparisons, and these features were also given high importance score in RF, indicating the effectiveness of these features for the prediction. Additionally, this was also supported by the results of the machine learning analysis, as we obtained the similar error rate compared to the lowest error rate using a combination of common “family” and “order” for the main dataset and the common “order” only for the mystery datasets. Furthermore, ANCOM analysis helped us to find the marker feature, in other words, even though *Flavobacteriaceae* existed in all the 16 cities of the main dataset, the relative abundance of *Flavobacteriaceae* in Sacramento was significantly different from most of the other cities. Therefore, *Flavobacteriaceae* could be used as the marker for identifying the samples from Sacramento. The members of the *Flavobacteriaceae* family are found in a wide variety of marine, freshwater, and soil habitats, and some are also associated with animals or plants [10]. In addition to the microbial data, some other city-specific data such as weather data could be considered including for future work, as microbial composition could be affected by different environments.

In summary, the results presented in this work showed an effective method to process, and classify the samples by origin, but there is still much to be improved, worth investigating in future work.

## Methods

The design of the analysis was motivated by the experience from the CAMDA 2017 and CAMDA 2018 MetaSUB Challenges [4, 11]. Compared to date from previous MeteSUB challenges, the data this year was with higher quality and deeper sequencing depth. As we have more cities included this year, the common features shared by all the cities were further limited. Therefore, the features selection was implemented this year to help us obtain the qualitied features for classification. Finally, unsupervised and supervised techniques were used for the analyses. A more detailed description of the implementations was supplemented in the following sections.

### Bioinformatics and data preparation

The Bioinformatics and data preparation was based on our previous paper [4]. Samples were Illumina-sequenced at different depths and delivered as FASTQ format for further analysis. Subsequent bioinformatics processing and data preparation were conducted in the “HyperGator2” high-performance cluster at the University of Florida. The supercomputer cluster includes 46,000 CPUs each with 4GB of RAM. Regarding the samples data processing, generally the bioinformatic part was not highly demanding of computer power, but OTU calling with QIIME required on an average approximately 500 CPU hours, running in 24 processors. This running time was largely driven by the size of the raw FASTQ files. Phred score filtering was implemented with FASTX-Toolkit (version 0.0.14 released Jan-05-2014) [12] for preliminary quality control, the parameters used in the filtering were q = 38 as a minimum Phred score to keep and *p* = 50 to set a minimum percentage of the bases that must have a quality score of 38. After quality control, samples with the poor sequencing depth were removed and the data was then transformed in a FASTA format for performing open reference OTU picking with QIIME with default setting [13]. After OTU picking, all the counts with mapping quality scores calculated by the RDP taxonomy classifier [14] < 0.5 were removed from further analyses. All further data processing and analyses in this study were conducted in R [15]. The R-code for the analyses will be available on https://susmdatta.github.io/software.html.

### Selecting common features

For the main dataset, the common features existing across all the 16 cities from a specific rank (“species”, “family” or “order”) were selected. To obtain more common features, two additional rules were implemented for the features from a specific rank: i) features existing in at least N cities were selected (Top features with the highest ubiquity across all the cities). N was set to 15, 14, 13, 12, 11, 10, 9 and 8 respectively, the count of the feature did not exist in the city was marked as zero. ii) features were reordered based on their ubiquity across all the samples, the top M features with the highest ubiquity were selected. M was set to 10, 20, 30, 50, 100, 150 respectively. In addition to using the selected feature sets of different ranks independently, the common features from different ranks were combined and regarded as the combined common features for the analyses. In other words, features from different ranks were used together as the input for the analyses. For example, the combined features with common “species” and “family” indicate that the common features from rank “species” along with the common features from rank “family” will be used together rather than independent for the analyses. The mystery dataset was analyzed based on the common features and combined features for simplicity. The data were then normalized to generate log2-cpm to make the data meaningful using the function “voom” [16] in R package “limma” [17] for further analysis.

### Machine learning analysis

Three classification algorithms were implemented at this stage for both the main and mystery datasets: Random Forest (RF) [5], Support Vector Machine (SVM) [6] and Linear Discriminant Analysis (LDA) [18]. Leave-one-out cross validation was conducted, the test sample was selected in order for each run with other samples served as the training set, and three methods were then implemented based on the same data. The results of prediction of three classifications for each sample was obtained. The overall error rate and the error rate for each city were calculated based on the results from each run. RF was conducted using the R package “randomForest”, 1000 trees were used and the count of variables chosen at each split was equivalent to the square root of the number of features in the dataset. The variable importance score [19] computed by the RF was also recorded. The SVM classifier was implemented using the R function “best.svm” in package “e1071” [20]. The two important parameters in SVM, i.e. gamma and c-value, affected the fitting of the models. The SVM model with the appropriate parameters was obtained by testing the performance of models with different parameters for each run independently. The LDA was conducted in a similar manner using the R package “MASS” [21].

When the models based on the main dataset were used to predict the labels of samples from mystery dataset. 50% of the samples of each city were randomly sampled from mystery dataset and added to the main dataset with the labels of “mystery” to serve as the part of the training dataset, the rest of the samples were served as the test samples. The sampling and the prediction were implemented for 1000 times. For each run, the predicted labels of test samples were checked with the real labels, a good prediction meant all test samples could be labeled as “mystery” by the classification.

### Principal coordinates analysis

Principal coordinates analysis (PCoA) [8] of normalized data was conducted using the R package “vegan” [22] and “ape” [23]. Firstly, the Bray-Cruits dissimilarity matrix was constructed. Then, the dissimilarity matrix was used for PCoA and a set of uncorrelated axes was generated to summarize the variability in the dataset. The two-dimensional plot was generated for assessing the separation of the cities.

### Analysis of composition of microbiomes

Analysis of composition of microbiomes for the normalized data was conducted using the R package “ANCOM” [9], which accounted for the underlying structure in the microbial data and was used for comparing the composition of microbiomes in two or more populations. For the main and mystery datasets, ANCOM across all pair-wise comparisons in each dataset was implemented. The 120 and 28 pair-wise comparisons were made for the combination of all cities in the main and mystery datasets respectively. And the output of the ANCOM was a set of differentially abundant features between the two cities for each comparison at the significance level of 0.05. The heatmap was made based on the output for investigating the difference of microbial composition between different cities. Additionally, the results of ANCOM were compared with the importance score derived by the RF method.

## Data Availability

The datasets supporting the conclusions of this article can be obtained from the CAMDA 2019 website http://camda2019.bioinf.jku.at/doku.php/contest_dataset.

## Abbreviations

NGS: Next Generation Sequencing
WGS: Whole genome sequencing
OTU: Operational Taxonomic Unit
RF: Random Forest
SVM: Support Vector Machine
LDA: Linear Discriminant Analysis
CV: Cross Validation
PCoA: Principal Coordinates Analysis
ANCOM: Analysis of composition of microbiomes
